# Identification of lipopolysaccharide-binding peptide regions within HMGB1 and their effects on subclinical endotoxemia in a mouse model

**DOI:** 10.1002/eji.201141391

**Published:** 2011-06-10

**Authors:** Ju Ho Youn, Man Sup Kwak, Jie Wu, Eun Sook Kim, Yeounjung Ji, Hyun Jin Min, Ji-Ho Yoo, Ji Eun Choi, Hyun-Soo Cho, Jeon-Soo Shin

**Affiliations:** 1 Department of Microbiology, Brain Korea 21 Project for Medical Science, Yonsei University College of MedicineSeoul, Korea; 2 Institute for Immunology and Immunological Diseases and Severance Biomedical Science Institute, Yonsei University College of MedicineSeoul, Korea; 3 NCRC for Nanomedical Technology, Yonsei University College of MedicineSeoul, Korea; 4 Department of Pediatrics, Seoul National University Borame HospitalSeoul, Korea; 5 Department of Biology, Yonsei UniversitySeoul, Korea

**Keywords:** Endotoxin shock, High mobility group box 1, Inflammation, Lipopolysaccharide

## Abstract

Lipopolysaccharide (LPS) triggers deleterious systemic inflammatory responses when released into the circulation. LPS-binding protein (LBP) in the serum plays an important role in modifying LPS toxicity by facilitating its interaction with LPS signaling receptors, which are expressed on the surface of LPS-responsive cells. We have previously demonstrated that high mobility group box 1 (HMGB1) can bind to and transfer LPS, consequently increasing LPS-induced TNF-α production in human peripheral blood mononuclear cells (PBMCs). We report here on the identification of two LPS-binding domains within HMGB1. Furthermore, using 12 synthetic HMGB1 peptides, we define the LPS-binding regions within each domain. Among them, synthetic peptides HPep1 and HPep6, which are located in the A and B box domains of HMGB1, bind to the polysaccharide and lipid A moieties of LPS respectively. Both HPep1 and HPep6 peptides inhibited binding of LPS to LBP and HMGB1, LBP-mediated LPS transfer to CD14, and cellular uptake of LPS in RAW264.7 cells. These peptides also inhibited LPS-induced TNF-α release in human PBMCs and induced lower levels of TNF-α in the serum in a subclinical endotoxemia mouse model. These results indicate that HMGB1 has two LPS-binding peptide regions that can be utilized to design anti-sepsis or LPS-neutralizing therapeutics.

## Introduction

Lipopolysaccharide (LPS) is the main cause of Gram-negative bacterial sepsis. LPS consists of a lipid A component, a sugar moiety that forms the core, and an *O*-polysaccharide of variable length [1]. When LPS is introduced into the bloodstream, LPS-binding protein (LBP) recognizes the LPS molecules and catalyzes the movement of LPS from LPS aggregates. LBP transfers LPS to CD14, which in turn transfers LPS to the TLR4-MD2 receptor. Recently, the crystal structure of the TLR4-MD2-LPS complex has been determined [2]. Although there are several proteins that bind LPS, LBP is the first key protein that initiates and amplifies the LPS-mediated pro-inflammatory process that results in fatal septic shock syndrome.

The nuclear protein high-mobility group box 1 protein (HMGB1) is involved in nucleosome stabilization, gene transcription, and neurite outgrowth [3]. HMGB1 can be actively or passively released into the extracellular space through acetylation [4], phosphorylation [5, 6], methylation [7], or cell necrosis [8]. HMGB1 can trigger inflammation [8] and is a late mediator of endotoxemia and sepsis in both animal models and humans [9–12]. Although HMGB1 is a well-known mediator of endotoxemia and a proinflammatory cytokine-like protein in vivo, purified recombinant HMGB1 only has weak in vitro proinflammatory activity, such as the induction of TNF-α production [13, 14]. HMGB1 can form highly inflammatory complexes with CpG DNA [15, 16] and IL-1β [17], suggesting that HMGB1 is necessary but not sufficient to induce inflammation [18]. Previously, we proposed that HMGB1 can interact with LPS and transfer LPS to CD14 to enhance LPS-mediated inflammation [14]; HMGB1 may transfer LPS to CD14 under the conditions where LBP is absent, such as in LBP-deficient mice [19], or where and when the level of HMGB1 is highly increased such as in Gram-negative bacterial infections [9].

In this study, we describe two HMGB1 synthetic peptides (of 12 tested) that can bind to LPS, namely HPep1 (HMGB1^3–15^) and HPep6 (HMGB1^80–96^); these peptides bind to the polysaccharide and lipid A moieties of LPS respectively. We demonstrate that both these LPS-binding peptides inhibit LPS binding to LBP, LBP-mediated LPS transfer to CD14, and cellular uptake of LPS in RAW264.7 cells. Both HPep1 and HPep6 inhibited LPS-induced TNF-α release in human peripheral blood mononuclear cells (PBMCs) and also decreased serum levels of TNF-α in a mouse model of subclinical endotoxemia, suggesting that these two LPS-binding peptides may have potential as antiseptic therapeutics.

## Results

### HMGB1 A and B box proteins bind to different moieties of LPS

**Figure 1:**
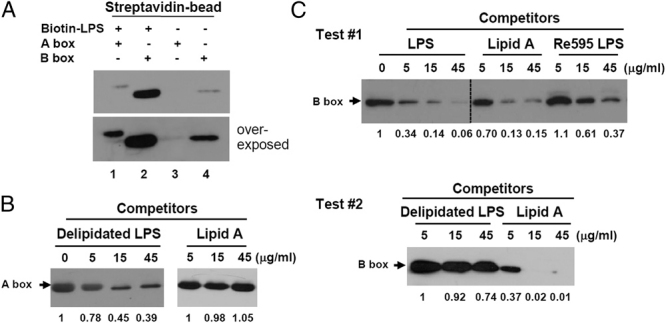
LPS-binding specificity of HMGB1 domains. (A) Biotin-labeled *E. coli* LPS was incubated with 6× His-tagged HMGB1 A and B box proteins and pull-down assays were performed. The beads were subjected to 12% SDS-PAGE and Western blot analysis was performed using anti-His Ab. (B, C) An aliquot of 5 μg/mL of biotin–LPS was incubated with 5 μg/mL of His-tagged A box or B box HMGB1 protein that had been preincubated with various amounts of *E. coli* delipidated LPS, *S. minnesota* lipid A, *S. minnesota* Re595 LPS, or WT *S. minnesota* LPS as inhibitors. Biotin–LPS was precipitated and analyzed using Western blotting with an anti-His Ab. (C) The line indicates the cutline of the same blot membrane. Data shown are representative of two independent experiments.

We previously found that HMGB1 can bind to LPS and transfer it to CD14 thereby enhancing LPS-mediated inflammation, demonstrating that HMGB1 plays a role in LPS-mediated TNF-α production [14]. In the current study, we further investigated the interaction between HMGB1 and LPS, and evaluated whether LPS-binding HMGB1 peptides can neutralize LPS. We first evaluated whether the A and B box domains of HMGB1 play a role in LPS binding. For this, 6-His-tagged HMGB1 A and B box proteins were produced in *Escherichia coli* [5] and incubated with biotin-tagged LPS for precipitation with streptavidin beads. The protein containing the HMGB1 B box domain bound very strongly to LPS, whereas the protein containing the A box domain bound weakly to LPS (Fig. 1A).

We next investigated which moiety of LPS – the polysaccharide or lipid A moiety – binds to the A and B box proteins of HMGB1. Biotin–LPS was incubated with a constant amount of HMGB1 A box protein in the presence of various amounts of partially delipidated LPS and lipid A as competitors, and the binding of A box protein to biotin–LPS was examined by Western blotting. The binding of the A box protein to biotin–LPS was inhibited by delipidated LPS although not completely inhibited due to its partial delipidation; however, lipid A did not inhibit the binding of the A box protein to LPS (Fig. 1B).

We next investigated the binding of the HMGB1 B box domain to LPS. When delipidated LPS and lipid A were added to the mixture of biotin–LPS and the HMGB1 B box protein, the binding of HMGB1 B box to biotin–LPS was inhibited by lipid A in a dose dependent manner, but not by delipidated LPS (Fig. 1C, upper). This inhibition was also observed using Re595 LPS and unlabeled WT LPS, both of which contain the lipid A moiety of LPS (Fig. 1C, lower). To predict the binding mode of HMGB1 and lipid A, we generated a model of HMBG1 and lipid A complex structure using molecular docking. The head region of lipid A is surrounded by the positive surface of HMGB1 box B (Supporting Information Fig. 1). Among four phosphate groups in the lipid A head and inner core regions of LPS, three bind to basic patches of HMBG1. The fatty acid tails of lipid A are forward to HMGB1 box A, forming weak hydrophobic interactions with nearby hydrophobic residues and suggesting that a major contribution of lipid A binding to HMGB1 is caused by the B box domain rather than by the A box.

These data demonstrate that the HMGB1 B box protein binds to the lipid A moiety of LPS. These results suggest that HMGB1 A and B box proteins bind to two different moieties of LPS, namely the delipidated polysaccharide and lipid A moieties respectively. These data are consistent with our previous data obtained using surface plasmon resonance analyses [14].

### Mapping of the LPS binding region of HMGB1

**Figure 2:**
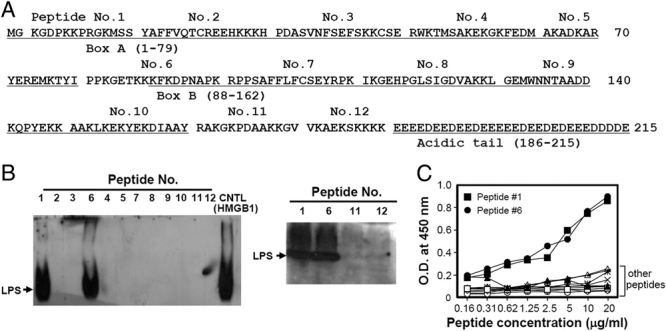
Mapping of the LPS-binding regions of HMGB1. (A) Twelve synthetic biotin-labeled HMGB1 peptides were prepared for the LPS binding study. Boxes A and B and the acidic tail domain are underlined. (B) Briefly, 10 μg/mL of each biotin-labeled HMGB1 peptide was incubated with 10 μg/mL of LPS. Pull-down assays were performed with streptavidin agarose beads and analyzed by Western blotting. The membrane was probed with an anti-LPS Ab. WT HMGB1 was used as a positive control (left). This assay was repeated using four selected peptides (right). (C) Microtiter plates were coated with 10 μg/mL of LPS in PBS and washed with 0.05% Tween-20 PBS. Various concentrations of each biotin-labeled HMGB1 peptide were added to the wells followed by the addition of HRP-conjugated streptavidin. TMB solution was used as a substrate for color development. Data shown are representative of two independent experiments.

To further investigate the binding of A and B box HMGB1 proteins to LPS, 12 biotin-labeled HMGB1 peptides were synthesized (Fig. 2A) and their LPS-binding properties analyzed. Given that both the LPS and the heparin-binding region have the motif BBXB, where B is any basic aa and X is any hydrophobic aa [20], the length of the peptides was constrained to preserve this motif. Each biotin-labeled peptide was incubated with LPS and precipitated with streptavidin beads. As shown in Fig. 2B (left and right panel), HMGB1 peptides No. 1 (HPep1, HMGB1^3–15^) and No. 6 (HPep6, HMGB1^80–96^) bound to LPS in contrast to the other ten peptides. We used an ELISA assay to confirm binding of only these two peptides to LPS. The biotin-labeled peptides were added to LPS-coated wells and HRP-conjugated streptavidin was added, and only HPep1 and HPep6 bound to LPS-coated wells in a dose-dependent manner (Fig. 2C).

### HPep1 and HPep6 bind to different moieties of LPS and inhibit binding of LPS to LBP and HMGB1

**Figure 3:**
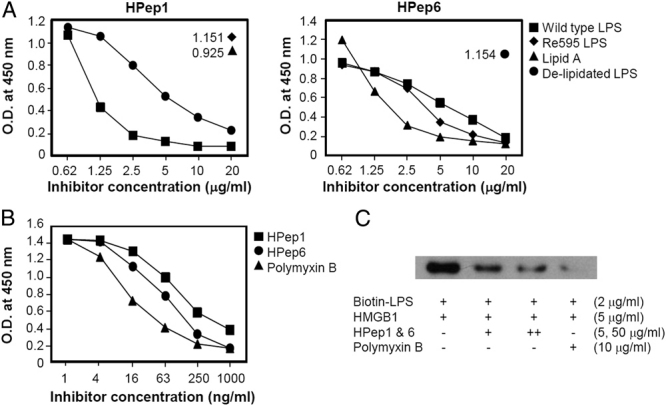
Binding of HMGB1 peptides to LPS. (A) Competitive binding analysis of the interaction between the HMGB1 peptides and LPS. Microtiter plates were coated with LPS, and the same amount of peptide No. 1 (HPep1) or No. 6 (HPep6) was added to the wells in the presence of various amounts of *S. minnesota* Re595 LPS, *S. minnetosa* lipid A, or *E. coli* delipidated LPS. WT *S. minnetosa* LPS was used as a positive control inhibitor. The binding of the HMGB1 peptides to LPS was probed by HRP-conjugated streptavidin. (B, C) Inhibition of LPS binding to LBP and HMGB1 by HMGB1 peptides. (B) Briefly, 100 ng/mL of LBP was added to LPS-coated wells in the presence of various amounts of HMGB1 peptides. The binding of LBP to LPS was probed using an anti-LBP Ab. Polymyxin B was used as a positive control inhibitor. (C) Biotin-labeled LPS was incubated with 5 μg/mL of HMGB1 in the presence of a mixture of HPep1 and HPep6 peptides, and pull-down assays were performed using streptavidin agarose beads. Western blot analysis was performed using anti-HMGB1 Ab. Data shown are representative of two independent experiments.

We next investigated whether HPep1 and HPep6 bind the polysaccharide and lipid A moieties of LPS respectively, because HPep1 contains an A box domain protein sequence, whereas HPep6 contains a B box HMGB1 protein sequence and a part of the linker region, respectively (Fig. 2A). A constant amount of biotin–HPep1 or biotin–HPep6 was added to LPS-coated wells in the presence of various concentrations of delipidated LPS, lipid A, or Re595 LPS as competitors, and the binding of each biotin–peptide to LPS-coated wells was probed with HRP-conjugated streptavidin. WT LPS was used as a positive control competitor. As shown in Fig. 3A, the binding of HPep1 to LPS was dose dependently inhibited by polysaccharide moiety-containing partially delipidated LPS and WT LPS. On the contrary, the binding of HPep6 to LPS was inhibited by lipid A-containing LPS, Re595 LPS, and WT LPS. Re595 LPS and lipid A showed no inhibition to HPep1 to LPS at the concentration of 20 μg/mL, and delipidated LPS also showed no inhibition of HPep6 to LPS. These results demonstrate that HPep1 and HPep6 bind to the polysaccharide and lipid A moieties of LPS respectively.

We next investigated whether HPep1 and HPep6 could inhibit the binding of LPS to LBP. For this, a constant amount of LBP was added to LPS-coated wells in the presence of various concentrations of HPep1 and HPep6. LPS binding to LBP (Fig. 3B) and HMGB1 (Fig. 3C) was dose dependently inhibited by HPep1 and HPep6, similar to the dose-dependent inhibition observed using the positive control inhibitor, polymyxin B.

### The HMGB1 B box protein catalyzes the movement of LPS from micelles to CD14

**Figure 4:**
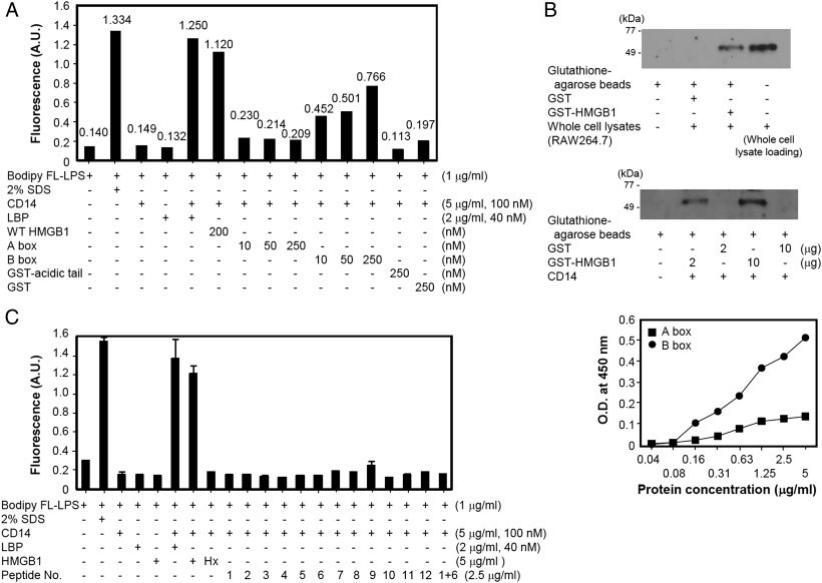
Ability of HMGB1 A- and B-box domain-containing peptides to transfer BODIPY FL-LPS to CD14. (A) A mixture of BODIPY FL-LPS and sCD14 was incubated in the presence of HMGB1 A box, B box, and GST-acidic tail proteins, and fluorescence levels were measured after 10 h at 25°C. LBP was used as a positive control, and 2% SDS was used to completely solubilize the disaggregated state of LPS for maximum fluorescence. (B) Interaction of HMGB1 with CD14. Either 2 or 10 μg of GST-HMGB1 protein was incubated with whole cell lysate from RAW264.7 cells (top) or recombinant CD14 protein (middle) and then precipitated with glutathione-Sepharose 4B beads. Western blot analysis was performed using an anti-CD14 Ab. Whole-cell lysate was loaded as a positive control. Binding of HMGB1 A and B box peptides to recombinant CD14 protein was measured by ELISA (bottom). A titration of 6-His-tagged A and B box proteins were added to the CD14-coated wells and anti-His Ab was used as the primary Ab. (C) HMGB1 peptide-mediated transfer of LPS to sCD14. In all, 1 μg/mL of BODIPY FL-LPS and 5 μg/mL of CD14 protein were incubated in the presence of 2.5 μg/mL of each HMGB1 peptide. Fluorescence levels were measured at 525 nm with a 488 nm excitation after 10 h at 25°C. LBP and HMGB1 proteins were used as positive controls. Heat-treated (Hx) HMGB1 was used as a control. Data shown are representative of three independent experiments. Error bars: standard deviation.

We next investigated which domain of HMGB1 catalyzes the fluorescence transition from an aggregated LPS state using BODIPY FL-LPS. BODIPY FL-LPS was incubated with soluble (s) CD14 in the presence of various concentrations of HMGB1 A and B box proteins, and the changes in fluorescence were measured. When BODIPY FL-LPS was incubated with WT HMGB1 in the presence of sCD14, the fluorescence level was 1.120, which is similar to that of BODIPY FL-LPS incubated with LBP (Fig. 4A) [14]. When HMGB1 A and B box proteins were tested, the level of fluorescence of BODIPY FL-LPS was dose dependently increased by the B box protein, but no detectable change in fluorescence was observed upon incubation with the A box protein. A fusion protein containing the GST-acidic tail of HMGB1 showed no change in fluorescence, confirming that the HMGB1 acidic tail plays no role in LPS catalysis [14].

We further investigated the binding of HMGB1 A and B box proteins to CD14. When GST-HMGB1 was incubated with whole-cell lysates of RAW264.7 cells as a CD14 protein source (Fig. 4B, top) or purified recombinant CD14 protein (Fig. 4B, middle) and then precipitated with glutathione–agarose beads, binding of CD14 was clearly observed, as expected. HMGB1 interacted with CD14 via the HMGB1 B box domain (Fig. 4B, bottom). Next, we investigated whether HPep1 and HPep6 could facilitate the fluorescence transition from an aggregated BODIPY FL-LPS state. However, treatment with HPep1 and/or HPep6 did not facilitate LPS transfer to CD14, although both these peptides bound to LPS (Fig. 4C), suggesting that the CD14-binding region is different from the LPS-binding regions of HMGB1.

### HPep1 and HPep6 inhibit LBP-mediated LPS transfer to CD14

**Figure 5:**
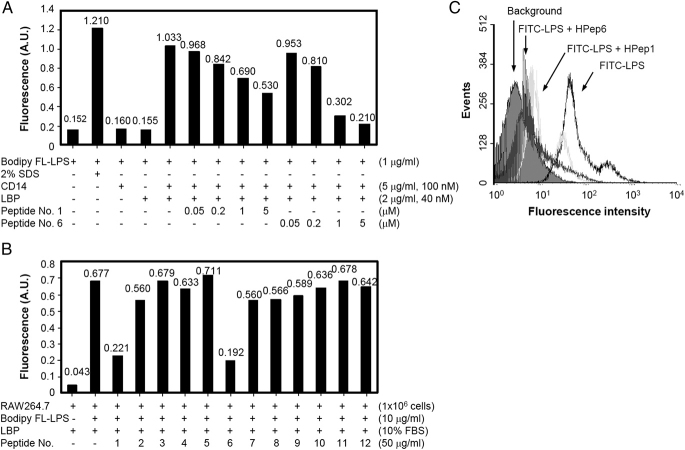
Inhibition of LPS transfer by HPep1 and HPep6. (A) A mixture of BODIPY FL-LPS, CD14 protein, and LBP was incubated in the presence of various amounts of HPep1 and HPep6, and changes in fluorescence levels were measured. (B) RAW264.7 cells were incubated with a mixture of BODIPY FL-LPS and LBP in the presence of each HMGB1 peptide and the fluorescence levels of RAW264.7 cells were measured after washing. (C) RAW264.7 cells were incubated with a preincubated mixture of 100 μg/mL of FITC-conjugated LPS and 100 μg/mL of HMGB1 peptide HPep1 or HPep6 in 10% FBS-DMEM. The binding of FITC-LPS was analyzed by flow cytometry after washing. Data shown are representative of two or three independent experiments.

Both HPep1 and HPep6 inhibited LPS binding to LBP and HMGB1 (Fig. 3B and C), and we next tested whether the next step of LPS transfer to CD14 molecule could be inhibited by these peptides. HPep1 and HPep6 were incubated with a constant amount of BODIPY FL-LPS and LBP in the presence or absence of sCD14. Both HPep1 and HPep6 inhibited the LBP-mediated transfer of BODIPY FL-LPS to sCD14 in a dose-dependent manner (Fig. 5A).

Next, we investigated whether HPep1 and HPep6 could also inhibit LBP-mediated LPS transfer at the cellular level. RAW264.7 cells were incubated at 37°C for 60 min with a preincubated mixture of BODIPY FL-LPS and LBP in the presence or absence of 50 μg/mL of each HMGB1 peptide, and the fluorescence produced by RAW264.7 cells was measured after washing. As shown in Fig. 5B, HPep1 and HPep6 significantly inhibited LBP-mediated LPS transfer to RAW264.7 cells.

We further analyzed whether HPep1 and HPep6 could inhibit the direct binding of LPS to RAW264.7 cells using flow cytometry. RAW264.7 cells were incubated with a preincubated mixture of FITC-LPS and HMGB1 peptide. When FITC-LPS was incubated with the same concentrations of HPep1 and HPep6, the mean fluorescence intensity (MFI) of FITC-LPS to RAW264.7 cells decreased from 69.8 (FITC-LPS only) to 12.0 and 8.4, respectively (Fig. 5C). These data indicate that HPep1 and HPep6 inhibit binding of LPS to RAW264.7 cells.

### HPep1 and HPep6 inhibit LPS-induced TNF-α production in human PBMCs

**Figure 6:**
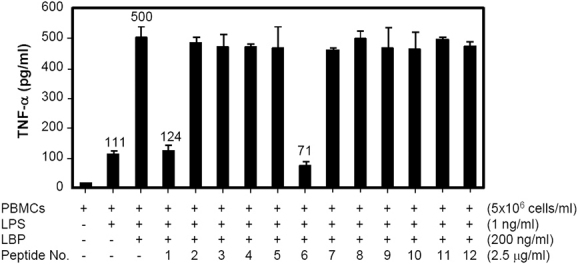
Inhibition of TNF-α production in human PBMCs by HMGB1 peptides. Human PBMCs (5×10^6^ cells/mL) were stimulated with a preincubated mixture of 1 ng/mL of LPS and 200 ng/mL of LBP in the presence of 2.5 μg/mL of each HMGB1 peptide in serum-free Opti-MEM® medium. The cultures were incubated 16 h at 37°C and the concentration of TNF-α in the culture supernatants was determined using sandwich ELISA. Data shown are mean and standard deviation of three independent experiments.

We next measured whether HPep1 and HPep6 had a neutralizing effect on LPS-induced TNF-α production in human PBMCs. Human PBMCs were treated with 1 ng/mL of LPS in the presence of 200 ng/mL of LBP and 2.5 μg/mL of each HMGB1 peptide for 16 h, and TNF-α in the culture supernatants was measured. When human PBMCs were treated with LPS in the absence of HMGB1 peptide, the mean level of TNF-α production was 500 pg/mL. TNF-α production decreased to 124 and 71 pg/mL after the addition of HPep1 or HPep6 respectively. Approximately 111 pg/mL of TNF-α was produced when human PBMCs were treated with 1 ng/mL of LPS without the addition of LBP. Other HMGB1 peptides did not inhibit LPS-induced TNF-α production. These data suggest that HPep1 and HPep6 function as LPS-neutralizing peptides (Fig. 6).

### HPep1 and HPep6 inhibit LPS-induced TNF-α production in a subclinical endotoxemia mouse model

**Figure 7:**
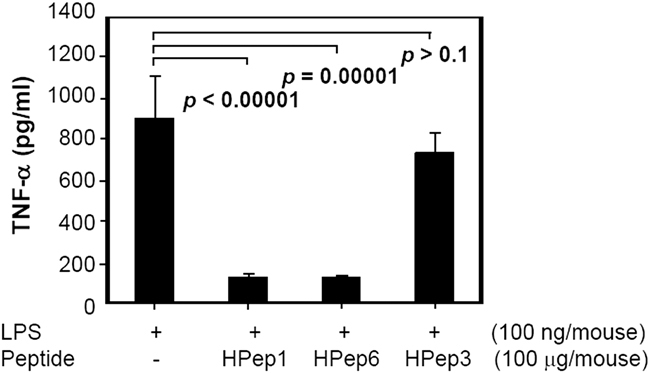
Neutralizing effect of the HMGB1 peptides HPep1 and HPep6 in a subclinical endotoxemia mouse model. BALB/c mice were injected intravenously with a subclinical dose of 100 ng of ultrapure *E. coli* LPS and 100 μg of HPep1 or HPep6. HPep3 (HMGB1 peptide No. 3), which does not bind to LPS, was used as a negative control. Six mice per group were evaluated. Serum samples were collected 2 h after injection and the serum concentration of TNF-α was determined using sandwich ELISA. Error bars of standard deviation are represented. Data shown are representative of two independent experiments. Dunn's test (nonparametric) was used for calculation of the *p*-values.

Finally, we tested whether HPep1 and HPep6 can inhibit LPS-induced TNF-α production in vivo using a mouse model. BALB/c mice (six mice per group) were injected intravenously with 100 ng of LPS in the presence or absence of 100 μg of HPep1 or HPep6, and serum samples were collected 2 h after LPS injection. HMGB1 peptide No. 3 (HPep3) was used as a negative control peptide. As shown in Fig. 7, the mean serum TNF-α level in the LPS-treated group was 903 pg/mL. However, in mice injected with LPS in combination with HPep1 or HPep6, serum TNF-α levels were reduced to 120 and 123 pg/mL respectively. The level of TNF-α in HPep3-treated mice was not significantly different from that in the LPS-injected group.

## Discussion

In a previous study, we demonstrated that HMGB1 is an LPS-binding molecule that can transfer LPS to CD14 and eventually to TLR4-MD2 receptors, thereby resulting in TNF-α production [14]. In the current study, we demonstrated that HMGB1 has two LPS-binding motifs located in its A and B box domains respectively, and that two synthetic peptides (HPep1 and HPep6) containing each of these LPS-binding motifs respectively, can inhibit LPS-stimulated TNF-α production both ex vivo in human PBMCs and in vivo in a mouse model of subclinical endotoxemia. Peptides derived from several LBPs, namely LBP, bactericidal/permeability-increasing protein, limulus anti-LPS factor [21], serum amyloid protein [22, 23], lactoferrin [24], cationic protein (CAP)18 [25], and CAP37 [26] antagonize LPS (review [27, 28]). To this list, we can now add the two novel HMGB1-derived peptides HPep1 and HPep6.

Lipid A is the main target of LPS-neutralizing peptides [29]. Lipid A-binding peptides contain basic residues that interact ionically with the phosphate head groups of lipid A [27]. LPS-binding peptides have the motif of BBXB (B: any basic aa, X: any hydrophobic aa) [30, 31], which is a known binding site for both LPS and heparin. Another LPS-binding motif is a tripeptide of BZB residues (B: any basic aa, Z: aromatic ring-containing aa); the aromatic residue in this position plays an important role in LPS binding as revealed by a mutation study [32]. The HMGB1-derived peptide HPep6 (PPKGETKKKFKDPNAPK), which binds to lipid A, has overlapping BBXB and BZB motifs, whereas HPep1 (KGDPKKPRGKMSS), which binds to the polysaccharide portion of LPS, has a BBXB motif. However, a peptide sequence analysis of 29 LPS- or lipid A-binding dodecapeptides, selected by biopanning phage peptide library assay, revealed that these peptides are remarkably diverse sequences and have a high average p*I* value of 11.42 [33]. All dodecapeptides had predicted helical contents ranged from 0.0 to 0.26 based on the predictions of helix content by Agadir algorithm (http://agadir.crg.es) [34, 35]. These peptides had remarkably diverse sequences and structural propensities not limited to an amphipathic α-helical structure although many natural linear short peptides adopt an α-helical structure, suggesting that the potential of an amphipathic structure of short peptides may not be the prerequisite of LPS-binding affinity [33]. The HPep1 and Hpep6 peptides have relatively high p*I* values of 10.46 and 10.01 respectively, but show very different aa sequences and predicted helical contents of 0.01 and 0.23 by Agadir algorithm respectively, suggesting different helical structures.

HPep1 inhibited LPS binding to LBP and LPS-mediated TNF-α production in human PBMCs and mice with subclinical endotoxemia, although it is not a lipid A-binding peptide. Deacylated LPS can antagonize LPS at multiple LPS–LBP-binding sites in the LPS recognition pathway [36, 37], suggesting that the polysaccharide moiety of LPS plays an important role in LPS recognition. HPep1 binding to the polysaccharide moiety of LPS showed a similar inhibition mechanism as that of deacylated LPS. This, to the best of our knowledge, is the first study to report that a polysaccharide moiety-binding peptide can neutralize the binding of LPS to LBP. Further investigation is required to elucidate how binding of HPep1 to the polysaccharide moiety of LPS can inhibit the LPS–LBP interaction. CD14 binding is necessary for LBP-mediated LPS transfer [38], and LBP has both an LPS-binding domain and an LPS-transfer domain [39]. The HMGB1 B box protein has both a lipid A-binding motif and can bind to CD14, allowing the transfer of BODIPY FL-LPS to CD14. On the contrary, the HMGB1 A box protein has a polysaccharide-binding motif and shows little or no binding to CD14, and can therefore not affect LPS transfer. These results indicate that the HMGB1 B box domain plays a major role in HMGB1-mediated LPS transfer and LPS-mediated proinflammatory function. We are now studying the CD14-binding region of the HMGB1 B box domain.

LPS-neutralizing peptides are attractive for potential therapeutic use. Treatment of mice with either HPep1 or HPep6 resulted in reduced LPS-induced TNF-α production and both peptides showed no hemolytic activity (data not shown). HPep1 and HPep6 inhibited binding of LPS to LBP and HMGB1 by binding to different target sites on LPS, and further evaluation is necessary to determine whether combined treatment with both peptides can inhibit LPS-mediated TNF-α production to a greater extent than treatment with each peptide alone. One of the main problems associated with peptide therapy is the short half-life of peptides in serum caused by proteolytic degradation, and therefore optimization of peptide stability for longer persistence in blood, for example a tetrabranched peptide, may be necessary [40]. The stability of HPep1 and HPep6 peptides in serum and their possible use as anti-microbial peptides need to be evaluated in the future studies.

In summary, HMGB1 contains domains with lipid A- and polysaccharide-binding motifs that can neutralize LPS-mediated TNF-α release in human PBMCs and in a subclinical endotoxemia mouse model; peptides that cover these binding regions may potentially be used as anti-septic therapeutic agents and contribute the survival rate for pathological condition of LPS-mediated sepsis.

## Materials and methods

### LPS, synthetic peptides, and recombinant proteins

WT LPS (*E. coli* 0111:B4), Remutant LPS (lipid A and the sole constituent of the core of 2-keto-3-deoxyoctonate from *Salmonella minnesota* Re595), delipidated LPS (*E. coli* 0111:B4), lipid A (*S. minnesota*), and biotin-labeled *E. coli* LPS were purchased from Sigma. Delipidated LPS is partially delipidated product by alkaline hydrolysis. Recombinant human LBP and soluble CD14 protein (sCD14, aa 1-352) were purchased from R&D. Recombinant human HMGB1, HMGB1 A (aa 1-17), and B (aa 88-162) box proteins were produced previously [5]. GST-HMGB1 and GST-acidic tail of HMGB1 (aa 186-215) constructs were subcloned and expressed in *E. coli* BL21. To map the LPS-binding region of HMGB1, 12 biotin-labeled HMGB1 peptides (HPep1–HPep12) were synthesized (Peptron, South Korea) (Fig. 2A). Biotin-free synthetic peptides of HMGB1 were used for biological assays of TNF-α production.

### ELISA and competition ELISA

LPS ELISAs were performed as described previously [14]. Microtiter plates (Corning) were coated with 1–10 μg/mL of LPS from *S. minnesota*, and blocked with 3% BSA-PBST. Each biotin-labeled HMGB1 peptide was added to the wells and incubated for 2 h at room temperature. HRP-conjugated streptavidin (R&D) was incubated for an additional 1.5 h. TMB solution was used for color development. To investigate the binding of HMGB1 A and B box domains to sCD14, various amounts of HMGB1 A and B box proteins were added to the wells coated with 10 μg/mL of sCD14 for 3 h at room temperature. Anti-His Ab was used as a primary Ab.

A competitive ELISA was performed to investigate the binding specificity of HMGB1 to LPS. A constant amount of biotin-labeled HMGB1 peptide was incubated in LPS-coated wells in the presence of various concentrations of lipid A, Re595, delipidated LPS, or WT LPS as inhibitors. WT LPS was used as a positive control inhibitor. The binding of HMGB1 peptide was probed by HRP-conjugated streptavidin. To investigate whether the HMGB1 peptides could inhibit the binding of LBP to LPS, 100 ng/mL of LBP was added to LPS-coated wells (5 μg/mL) in the presence of various amounts of HMGB1 peptides. The binding of LBP to LPS was probed using a polyclonal anti-LBP Ab (Abcam). Polymyxin B (USB) was used as a positive control inhibitor.

### Pull-down assays and Western blotting analysis

To analyze the binding of HMGB1 A and B box proteins to LPS, 10 μg/mL of biotin-labeled LPS (*E. coli*, Molecular Probes) was incubated with 10 μg/mL of HMGB1 A and B box proteins and a pull-down assay was performed using 50 μg/mL (50% slurry) streptavidin agarose beads (Pierce). The beads were washed and subjected to 12% SDS-PAGE followed by the transfer of proteins to a nitrocellulose membrane. Western blot analysis was performed using an anti-His Ab (Qiagen) and HRP-labeled goat anti-rabbit Ig as primary and secondary Abs, respectively.

To investigate which moieties of LPS the A and B box domains of HMGB1 recognize and bind to, a competition assay was performed. An aliquot of 5 μg/mL of biotin–LPS was incubated for 6 h with 5 μg/mL of His-tagged A box or B box HMGB1 protein that had been preincubated with various amounts of inhibitors (delipidated LPS, lipid A, Re595 LPS, or WT LPS) for 1 h at 4°C. Biotin–LPS was precipitated using streptavidin agarose beads and analyzed using a Western blot assay with anti-His Ab.

To investigate the binding of HMGB1 to LPS, 10 μg/mL of each biotin-labeled HMGB1 peptide was incubated with 10 μg/mL of LPS (*E. coli* and *S. enterica* serotype typhimurium) for 4 h. His-tagged HMGB1 protein (30 μg/mL) was used as a positive control. The pull-down complexes using streptavidin agarose beads were separated by 12% SDS-PAGE and analyzed by Western blotting with anti-LPS Ab (Hycult Biotech). To identify HMGB1 binding to CD14, RAW264.7 cells were lysed with a protease inhibitor cocktail. Cell homogenates were centrifuged at 20 000×*g* for 15 min and precleared by incubating with glutathione-Sepharose 4B beads at 4°C for 30 min. The precleared extracts (500 μg) were incubated with 2 μg/mL of GST-HMGB1 or GST immobilized on glutathione-Sepharose 4B beads for Western blotting. The membrane was blotted with anti-CD14 Ab (R&D).

### Measurement of BODIPY FL-LPS transfer to CD14 by fluorescence

LPS transfer to sCD14 was measured using the disaggregation method of BODIPY FL-LPS [14]. Briefly, 1 μg/mL of BODIPY FL-LPS (Molecular Probes) and 5 μg/mL of sCD14 protein were added to Ca^++^ and Mg^++^-free PBS in the presence of recombinant HMGB1 A or B box protein or each HMGB1 peptide. Human LBP protein was used as a positive control. The fluorescence levels were measured at 525 nm at an excitation wavelength of 488 nm after 10 h at 25°C. Complete disaggregation of BODIPY FL-LPS by 2% SDS was measured. To investigate LPS transfer at the cellular level, 1×10^6^ RAW264.7 cells were incubated in 10% FBS-containing medium with BODIPY FL-LPS in the presence of each peptide for 60 min at 37°C. The cell-associated fluorescence was measured after washing by a spectrofluorometer.

### Flow cytometric analysis of FITC-conjugated LPS to RAW264.7 cells

FITC-conjugated LPS (100 μg/mL, Sigma) was preincubated for 30 min at 25°C with each HMGB1 peptide at a concentration of 100 μg/mL in 10% FBS-DMEM. The mixture was then added to 5×10^5^ RAW264.7 cells and further incubated for 30 min at 25°C. The cells were washed three times with cold PBS and fixed in 1% paraformaldehyde solution. The binding of FITC-LPS was analyzed by LSRII flow cytometry (BD). The mean fluorescence intensity (MFI) was measured.

### TNF-α production

Human PBMCs were isolated from the blood of normal subjects by Ficoll-hypaque gradient centrifugation after obtaining the permission of the IRB (4-2007-0059), and cultured in serum-free Opti-MEM® medium (Invitrogen) at 5×10^6^ cells/mL in 96-well plates [14]. PBMCs were treated with a mixture of 1 ng/mL of LPS and 2.5 μg/mL of each HMGB1 peptide; the peptides were preincubated for 30 min at 37°C with 200 ng/mL of LBP. The cultures were incubated for 16 h at 37°C and the culture supernatants were collected after centrifugation. The concentration of TNF-α was determined using a sandwich ELISA assay (R&D).

### Induction of subclinical endotoxemia in a mouse model

To evaluate whether 2 of the 12 HMGB1 peptides had a neutralizing effect, BALB/c mice (6–8 wk) were intravenously injected with a subclinical dose of 100 ng of LPS (Ultrapure *E. coli* LPS, Invitrogen) in the presence of 100 μg of HMGB1 HPep1 or HPep6 after obtaining the permission of the animal IRB of our institution (approval No. 08-151). HPep3, which does not bind to LPS, was used as the negative control peptide. Serum samples were collected 2 h after injection. The levels of TNF-α in mouse serum samples were determined using a sandwich ELISA (R&D) after diluting the serum samples 1:10.

### Statistical analysis

Dunn's test (nonparametric) was implemented in SAS 9.1 to analyze the data of subclinical endotoxemia in a mouse model.

## Abbreviations:

HMGB1: high mobility group box 1 protein
LBP: LPS-binding protein
S: soluble
