# Supplementary material for: Identification of lipopolysaccharide-binding peptide regions within HMGB1 and their effects on subclinical endotoxemia in a mouse model
Source: Eur J Immunol. 2011 Jun 10;41(9):2753–62. doi: 10.1002/eji.201141391 (PMC3193378; doi:10.1002/eji.201141391)
Supplement: Supplementary file 1 [file eji0041-2753-SD1.pdf]

European Journal of Immunology

Supporting Information

for

DOI 10.1002/eji.201141391

Identification of lipopolysaccharide-binding peptide regions within HMGB1 and their effects on subclinical endotoxemia in a mouse model

Ju Ho Youn, Man Sup Kwak, Jie Wu, Eun Sook Kim, Yeounjung Ji, Hyun Jin Min, Ji-Ho Yoo, Ji Eun Choi, Hyun-Soo Cho and Jeon-Soo Shin

© 2011 WILEY-VCH Verlag GmbH & Co. KGaA, Weinheim

Supporting information

Identification of lipopolysaccharide-binding peptide regions within HMGB1 and their effects on subclinical endotoxemia in a mouse model

Ju Ho Youn, Man Sup Kwak, Jie Wu, Eun Sook Kim, Yeounjung Ji,

Hyun Jin Min, Ji-Ho Yoo, Ji Eun Choi, Hyun-Soo Cho, and Jeon-Soo Shin

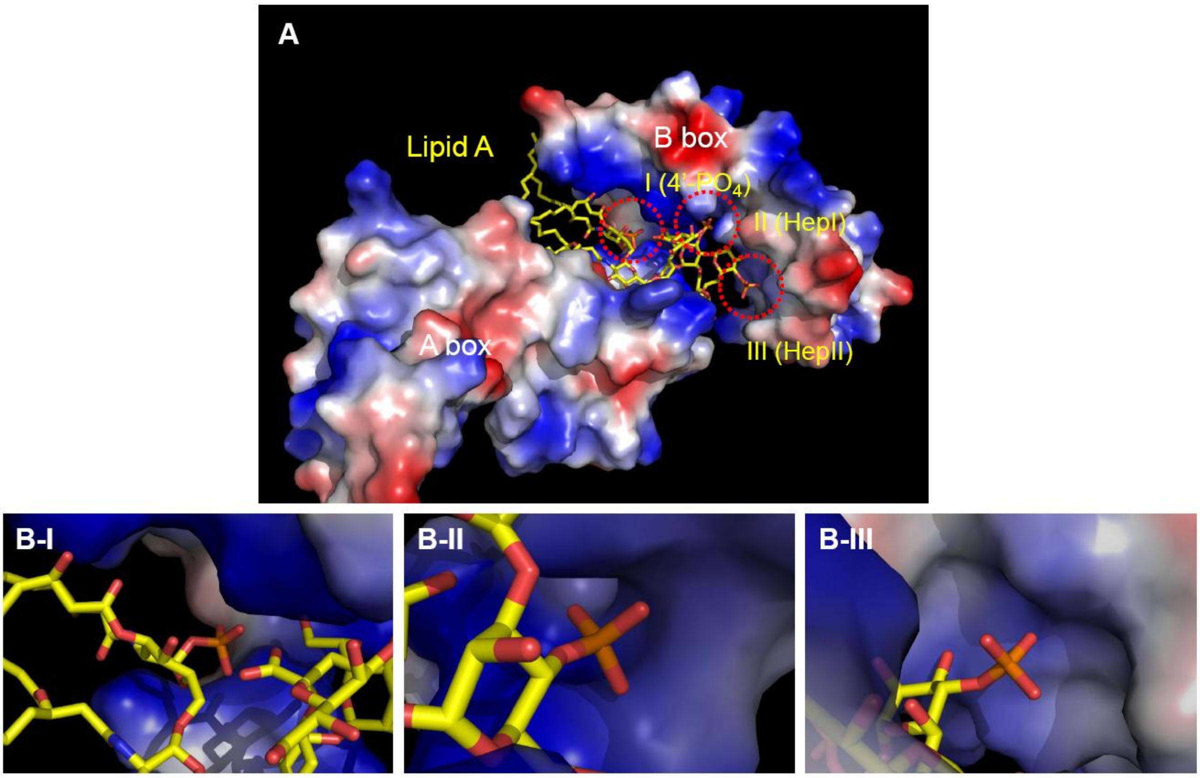

Supporting information Figure 1. Molecular docking model of lipid A binding to HMGB1. To build the HMGB1-lipid A complex model, we used the Patchdock server [1] with HMBG1 structure (PDB ID: 2YRQ) and lipid A structure from TLR4-MD structure [2] (PDB ID: 3FXI). According to the results of Patchdock server, we selected the top 10 models in scores and refined the models using the Firedock server [3-4]. Considering the global binding energy, the model with the highest score was chosen, which shows the most complementary shape. According to the model in Fig. 1A, the head region of lipid A (yellow color) is surrounded by a positive surface of HMGB1 box B. Blue color: basic residues, red color: acidic residues. Among the four phosphate groups in lipid A head (4'-PO_4 and 1-PO_4) and inner core (HepI and HepII) regions of LPS, three bind to basic patches of HMBG1 via electrostatic interaction and hydrogen bonds. Fig. 1B-I, -II, and -III are the magnified pictures of dotted circles of I, II, III, respectively.

References

1. **Schneidman-Duhovny, D., Inbar, Y., Nussinov, R., Wolfson, H.J.,** PatchDock and SymmDock: servers for rigid and symmetric docking. *Nucleic Acids Res* 2005. **33:**W363-367.
2. **Park, B.S., Song, D.H., Kim, H.M., Choi, B.S., Lee, H., Lee, J.O.,** The structural basis of lipopolysaccharide recognition by the TLR4-MD-2 complex. *Nature* 2009. **458:** 1191-1195.
3. **Andrusier, N., Nussinov, R., Wolfson, H.J.,** FireDock: fast interaction refinement in molecular docking. *Proteins* 2007. **69:** 139-159.
4. **Mashiach, E., Schneidman-Duhovny, D., Andrusier, N., Nussinov, R., Wolfson, H.J.,** FireDock: a web server for fast interaction refinement in molecular docking. *Nucleic Acids Res* 2008. **36:** W229-232.
